# What is the minimal dose for resistance exercise effectiveness in prostate cancer patients? Systematic review and meta-analysis on patient-reported outcomes

**DOI:** 10.1038/s41391-020-00301-4

**Published:** 2020-11-20

**Authors:** Pedro Lopez, Dennis R. Taaffe, Robert U. Newton, Laurien M. Buffart, Daniel A. Galvão

**Affiliations:** 1 Exercise Medicine Research Institute, Edith Cowan University, Perth, WA Australia; 2 School of Medical and Health Sciences, Edith Cowan University, Perth, WA Australia; 3 School of Human Movement and Nutrition Sciences, University of Queensland, Brisbane, QLD Australia; 4 Department of Physiology, Radboud University Medical Center, Radboud Institute for Health Sciences, Nijmegen, the Netherlands

**Keywords:** Prostate cancer, Cancer

## Abstract

**Background:**

Active treatments for prostate cancer are well known to result in several adverse effects such as fatigue, depression and anxiety symptoms, impacting the overall quality of life (QoL) and wellbeing of a considerable proportion of patients. Resistance-based exercise interventions have shown positive effects to reduce or mitigate these treatment-related side effects. However, the minimal dosage required to derive these benefits is unknown. We systematically reviewed the resistance training effects in prostate cancer patients to determine the minimal dosage regarding the exercise components (mode, duration, volume and intensity) on fatigue, QoL, depression and anxiety.

**Methods:**

Using PRISMA guidelines, MEDLINE, CINAHL, EMBASE, SPORTDiscus and Web of Science databases were searched. Eligible randomised controlled trials examined prostate cancer patients undertaking resistance-based exercise programs during or following treatment. Meta-analysis was undertaken when more than three studies were included. Associations between resistance exercise components and its effects were tested by meta-regression analysis.

**Results:**

Eighteen trials involving 1112 men with prostate cancer were included. Resistance-based exercise programs resulted in significant effects on fatigue (effect size = −0.3, 95% CI: −0.4 to −0.2, *P* < 0.001) and QoL (effect size = 0.2, 95% CI: 0.0 to 0.4, *P* = 0.018), with significant effects in specific questionnaires and domains of these outcomes. Resistance-based exercise effects on depression (effect size = −0.3, 95% CI: −0.7 to 0.0) and anxiety symptoms (effect size = −0.3, 95% CI: −0.5 to 0.0) were positive but not significant (*P* = 0.071 to 0.077). Meta-regression indicated no significant association between resistance exercise components with fatigue and QoL outcomes (*P* = 0.186–0.689).

**Conclusions:**

Low volume resistance exercise undertaken at a moderate-to-high intensity is sufficient to achieve significant fatigue and QoL benefits for men with prostate cancer and also mitigate depression and anxiety symptoms. A lower resistance exercise dosage than usually prescribed may help enhance adherence by reducing exercise barriers.

## Introduction

Several treatments are used to delay cancer progression and enhance survival such as androgen deprivation therapy (ADT) and radiotherapy in men with prostate cancer [1]. However, most patients are likely to face an array of treatment-related adverse effects during and after the course of treatment [2]. Among them, fatigue, depression and anxiety symptoms affect ~20 to 40% of men with prostate cancer undergoing primary treatment (e.g. surgery, radiotherapy and ADT), impacting their overall quality of life and wellbeing during and even following treatment [3–5].

Over the past decade, a substantial number of exercise trials have reported significant benefits to quality of life and fatigue in prostate cancer patients during ADT or following treatment when undertaking resistance-based exercise [6–9] with some of them also presenting modest changes in depression and anxiety symptoms [10, 11]. In the most recent exercise guideline for cancer patients [12], a resistance exercise prescription of two sets of 8–15 repetitions at 60–85% of one-repetition maximum (1-RM) in combination with aerobic exercise was recommended to counter anxiety, fatigue and depressive symptoms. However, most work underlying this recommendation was derived from trials in breast cancer patients and survivors, and there is a paucity of comparative trials regarding the resistance training components. As a result, it is unclear if this would be the most appropriate recommendation, especially in prostate cancer patients. This may be especially the case as to date there has only been a single trial comparing different resistance training frequencies in this patient group [13]. The study of Norris et al. [13] compared resistance training undertaken twice or three times per week for 12 weeks in prostate cancer survivors and did not observe any differences in quality of life, fatigue, depression or anxiety symptoms. The authors suggested that twice weekly training may be sufficient to provide benefits in these outcomes given the time constraints and possible exacerbation of symptoms such as fatigue related to undertaking exercise 3 days per week [13]. Although a promising result, it remains unknown if a resistance training volume and intensity, even lower than that previously suggested [12], may represent a minimal and sufficient stimulus for improvements in patient-reported outcomes in prostate cancer patients at different treatment stages. This is important given the role of resistance exercise to counter treatment-related toxicities in men with prostate cancer [2, 12]. Furthermore, despite several systematic reviews examining exercise effects in prostate cancer patients [14–19], none have addressed the minimal exercise dosage required for improvements in common patient-reported outcomes.

As a result, the aim of this review was to: (1) systematically review and analyse the resistance-based training effects on fatigue, quality of life, depression and anxiety outcomes in men with prostate cancer given their importance for patient wellbeing; and (2) examine the dose-response relationship between the prescribed exercise components (i.e. mode, duration, volume and intensity) and responses on those outcomes.

## Methods

### Study selection procedure

The systematic review was undertaken in accordance with the Preferred Reporting Items for Systematic Reviews and Meta-Analyses (PRISMA) statement [20, 21]. Furthermore, the method used was based on the minimum criteria established by the Cochrane Back Review Group (CBRG) [22].

This review included published data from randomised controlled trials evaluating the effects of supervised resistance-based exercise programs in prostate cancer patients at any treatment stage (including post treatment). The primary outcome for this review was fatigue, with secondary outcomes of quality of life, depression and anxiety. The exclusion criteria were: (1) home-based exercise as the only intervention during the intervention period due to lack of direct supervision and inability to quantify training variables (e.g. kilograms used as a resistance with resistance training machines or dumbbells in clinic-based programs compared to elastic bands or bodyweight often used in home-based programs); (2) trials involving mixed cancer patients without specific information on the results for prostate cancer patients; (3) trials not including or reporting on the specific outcomes for this review, or did not include sufficient information for analysis (e.g., baseline and post-intervention assessment, or within- and between-groups mean difference); and (4) written in a language other than English. In the search strategy, titles and abstracts were first independently evaluated. When abstracts did not provide sufficient information, they were selected for full-text evaluation. Eligibility was assessed independently by two reviewers, with differences resolved by consensus.

We included publications up to November 2019 using the following electronic databases: MEDLINE, CINAHL, EMBASE, SPORTDiscus and Web of Science. The terms used were: ‘prostate cancer’ and ‘resistance training’ in association with a list of sensitive terms (Supplementary Material Table S1). In addition, we also performed a manual search of the reference lists provided in the selected papers as well as previous systematic reviews and meta-analytic studies in patients with prostate cancer [14–19] to detect studies potentially eligible for inclusion.

### Data extraction

The data extraction was performed via a standardised form. Clinical information of the patients such as age, disease stage and treatment phase and intervention characteristics that included duration, components of resistance training such as prescribed modality, frequency, intensity and volume, adherence (i.e. number of patients that completed the program), attendance (i.e. number of sessions attended), compliance (i.e. number of patients that successfully completed the exercise prescription) and adverse events were extracted along with the main outcomes. Information was always extracted for the longest period of the supervised exercise intervention, while outcomes were extracted in their absolute units (e.g., questionnaire scores).

### Assessment of risk of bias

The risk of bias was evaluated according to the 2nd version of the Cochrane risk-of-bias tool for randomised trials (RoB 2) [23] with each assessment focused at the outcome level. The six-domain instrument includes: (1) randomisation process; (2) deviation from intended interventions; (3) missing outcome data; (4) measurement of the outcome; (5) selection of the reported result and (6) overall bias. Overall risk of bias was expressed as “low risk of bias” if all domains were classified as low risk, “some concerns” if some concern was raised in at least one domain but not classified as at high risk in any other, or “high risk of bias” if at least one domain was classified as high risk, or have multiple domains with some concerns [23].

### Data analysis

For the meta-analysis, the pooled effect estimates were obtained from the standardised mean difference (SMD) combining different questionnaire scores for the same respective outcome, and mean difference (MD) for each individual questionnaire, of baseline to the final assessment corresponding to the period of the intervention. Analyses were conducted for all studies and a subgroup analysis was provided for low risk randomised controlled trials based on RoB 2.0 when more than three studies were available. Fatigue was assessed using the following instruments: the Functional Assessment of Cancer Therapy—Fatigue (FACT-F) [24], European Organisation for Research and Treatment of Cancer Quality of Life Questionnaire C30—Fatigue (EORTC QLQ-C30_Fatigue_) [25], Multidimensional Fatigue Symptom Inventory-Short Form (MFSI-SF) [26]*,* Functional Assessment of Chronic Illness Therapy (FACIT) [27], Brief Fatigue Inventory (BFI) [28], and the Schwartz Cancer Fatigue Scale [29] questionnaires. The Functional Assessment of Cancer Therapy—Prostate (FACT-P) and—General (FACT-G) [30], the 36-Item Short-Form Health Survey (SF-36) physical and mental health composite [31] and EORTC QLQ-C30—Global [32] were used to assess quality of life. The Center for Epidemiologic Studies (CES-D) [33] and Brief Symptom Inventory-18—Depression (BSI-18_Depression_) were used to assess depression, and the BSI-18_Anxiety_ [34] and Memorial Anxiety Scale for Prostate Cancer (MAX-PC) [35] to assess anxiety. The questionnaires with their respective characteristics (number of items, scaling and scores), minimally important difference (MID) and cut-off points are described in Table 1. In questionnaires reverse scaled for fatigue, depression and anxiety outcomes, where higher values indicate better outcomes rather than poorer outcomes, the mean in each group was multiplied by −1 as recommended in the Cochrane Handbook [36].

**Table 1:** Patient reported outcome questionnaires for fatigue, quality of life, depression and anxiety.

Questionnaire	Items and scaling	Score	MID	Cut-off point
Fatigue				
FACT-F [24]	13 items; 5-point Likert rating scale	52 High—Less fatigue	3 pts [67]	34 pts [68]
FACIT-Fatigue [27]	13 items; 4-point Likert rating scale	52 High—Less fatigue	NR	43 pts [69]
MFSI-SF [26]	30 items; 4-point Likert rating scale	72 High—More fatigue	NR	NR
BFI [28]	9 items; 11-point Likert rating scale	10 High—More fatigue	NR	NR
Schwartz Cancer Fatigue Scale [29]	28 items; 5-point Likert rating scale	36 High—More fatigue	5 pts [70]	NR
EORTC QLQ-C30_Fatigue_ [25]	3 items; 4-point Likert rating scale	100 High—More fatigue	5 pts [71]	NR
Quality of life				
FACT-G [30]	27 items; 5-point Likert rating scale	100 High—Better QoL	4 pts [67]	61.3 pts [72]
FACT-P [30]	12 items; 5-point Likert rating scale	48 or 148^a^ High—Better QoL	NR	76.0 pts [73]
EORTC QLQ-C30 [32]	4- and 7-point Likert rating scale	100 High—Better QoL	5–10 pts—small change; 10–20 pts—moderate change; >20 pts—large change [74, 75]	70.0 pts [76]
SF-36 [31]	36 items; 3-, 5- and 6- point Likert rating scale	100 High—Better QoL	5 pts [77]	NR
Depression				
CES-D [33]	20 items; 4-point Likert rating scale	60 High—Greater depressive symptoms	NR	NR
BSI-18_Depression_ [34]	18 items; 5-point Likert rating scale	24 High—Greater depressive symptoms	NR	NR
Anxiety				
BSI-18_Anxiety_ [34]	18 items; 5-point Likert rating scale	24 or T-scores High—Greater depressive symptoms	NR	NR
MAX-PC [35]	24 items; 4-point Likert rating scale	72 High—Greater anxiety symptoms	NR	NR

*BFI* Brief Fatigue Inventory, *BSI-18* Brief Symptom Inventory-18, *CES-D* Center for Epidemiologic Studies—Depression Scale, *EORTC QLQ-C30* European Organisation for Research and Treatment of Cancer Quality of Life Questionnaire C30, *FACIT* Functional Assessment of Chronic Illness Therapy, *FACT-F* Functional Assessment of Cancer Therapy—Fatigue, *FACT-G* Functional Assessment of Cancer Therapy—Genera, *FACT-P* Functional Assessment of Cancer Therapy—Prostate, *MAX-PC* Memorial Anxiety Scale for Prostate Cance, *MID* minimally important difference, *MFSI-SF* Multidimensional Fatigue Symptom Inventory-Short Form, *NR* not reported elsewhere, *SF-36* 36-Item Short-Form Health Survey.

^a^Score based in the sum of FACT-G general score and the prostate cancer subscale.

In studies with multiple exercise interventions vs. a single control group, data from exercise groups was combined according to the Cochrane Handbook [36]. Calculations were performed using a random-effects model with the DerSimonian & Laird method [37]. Statistical significance was assumed when the mean difference effect reached an α value ≤0.05. Effect size (ES) were according to Cohen [38] with values of 0.0 to <0.5 indicating small, values of 0.51 to <0.8 indicating medium, and values ≥0.8 indicating large effects. Statistical heterogeneity was assessed using the Cochran Q test [39]. A threshold *P* value of 0.1, as well as values greater than 50% in I² were considered indicative of high heterogeneity [39]. We examined heterogeneity using sensitivity analysis by omitting one study at a time. Publication bias was explored by contour-enhanced funnel plots and Egger’s test [40], and, if necessary, trim-and-fill computation was used to estimate the effect of publication bias on the interpretation of results [39, 41]. Analyses were conducted using the package *metan*, *confunnel, metabias*, and *metatrim* from Stata 14.0 software (Stata, Texas, USA). Forest plots presented for the outcome measures are after sensitivity analysis and/or trim-and-fill procedure adjustments.

In addition, we tested the association between exercise components (mode, intervention duration, prescribed weekly volume and peak intensity) and SMD effects to identify a dose-response relationship. Using one or multiple variables at a time we assessed whether exercise components influence the association of resistance-based exercise with the main effects. Analyses were undertaken in outcomes significantly affected by exercise provided the models had more than 5 studies. For intervention duration, prescribed weekly volume and peak intensity, analyses were considered when the range was higher than 5%, while exercise mode was coded as 0 = resistance training alone and 1 = resistance training combined with other components (e.g. aerobic, flexibility, impact-loadingor balance). Correlations were weighted by the inverse of the variance of each observation and the coefficient of determination (r^2^), the statistical test of heterogeneity (I^2^), component coefficients, standard errors (SE) and 95% CI are presented for each outcome with their respective *P* values. Analyses were conducted using the package *metareg* from Stata 14.0 software (Stata, College Station, USA).

## Results

### Studies included

**Fig. 1:**
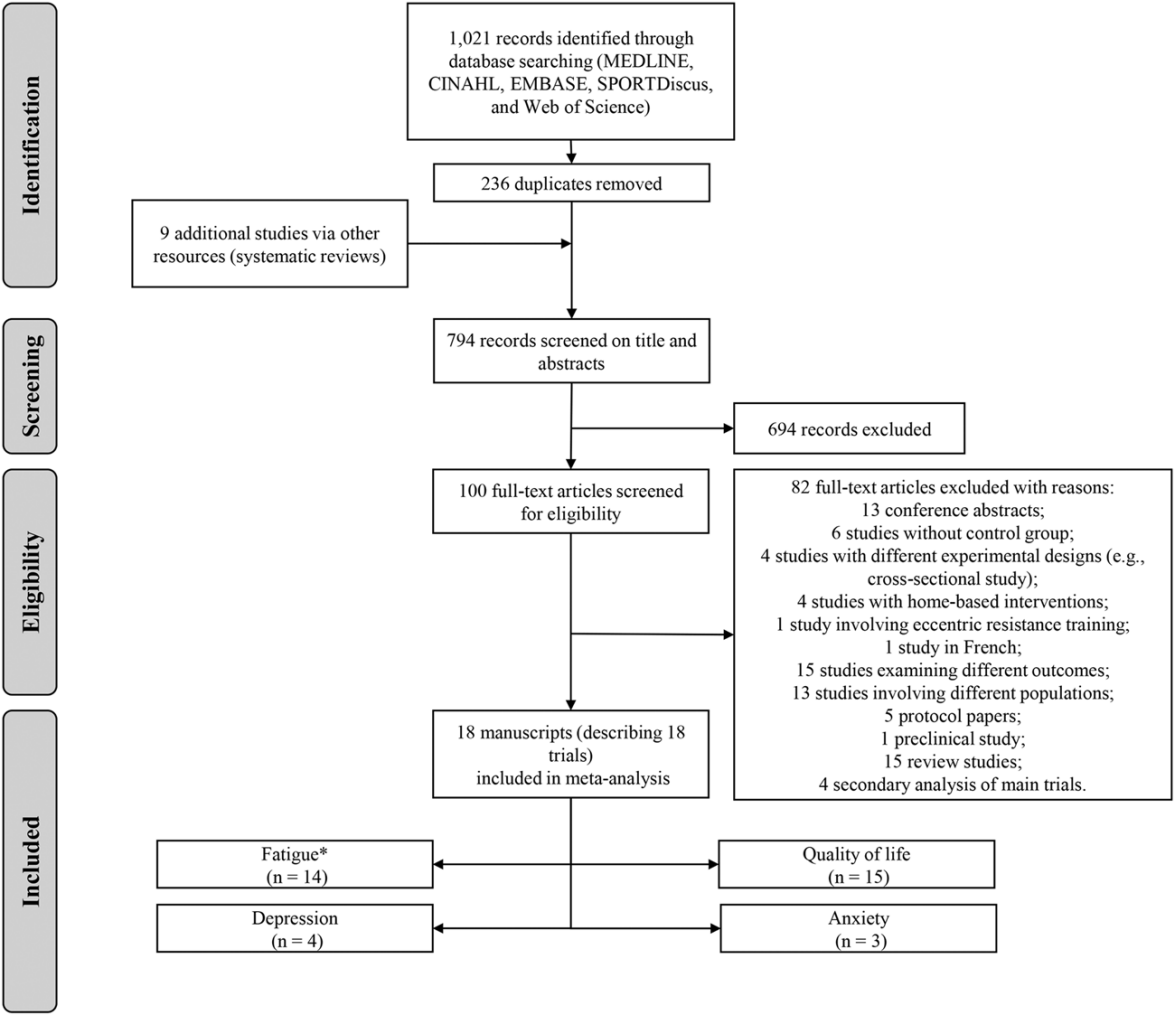
Flow chart of study selection process. Asterisk indicates primary outcome.

Of the 1030 retrieved studies, 794 were retained for screening after duplicate removals. Of these, 694 were excluded and 100 full-text articles were assessed for eligibility (Fig. 1) in accordance with the inclusion criteria. The eligibility assessment resulted in 18 [refs. 6–8, 10, 11, 42–54] studies that investigated the effect of resistance-based training (i.e. resistance training alone, combined with aerobic exercise or included in a multimodal exercise program) on patient-reported outcomes in prostate cancer patients at any treatment stage. Sixteen studies were included in the dose-response relationship analysis involving exercise mode, intervention duration, prescribed weekly volume and peak intensity and the effects on patient-reported outcomes.

### Prostate cancer patients and exercise intervention characteristics

A total of 1112 prostate cancer patients with an average age of 69.6 ± 2.2 yrs participated in the included studies. Exercise interventions were predominantly undertaken in patients on ADT (14 of 18 studies) [6, 8, 10, 11, 42–44, 46–48, 50, 52–54]. Exercise modalities included predominantly combined resistance and aerobic training (8 of 18 studies) [6–8, 10, 44, 46, 50, 54], followed by multimodal exercise programs (6 of 18 studies) [8, 11, 47, 49, 51, 53], and resistance training only (5 of 18 studies) [42, 43, 45, 48, 52] in a cohort of 555 patients allocated to exercise intervention compared to 557 patients in the control group. A total of 13 studies [6, 10, 11, 42–46, 48–51, 54] compared an exercise intervention vs. usual care control, 4 [refs. 7, 47, 52, 53] were compared to a home-based intervention involving aerobic or flexibility training or to physical activity advice, and 1 [ref. 8] was compared to a delayed exercise group. Studies characteristics are shown in Table 2.

**Table 2:** Characteristics of included studies reporting fatigue, quality of life, depression and anxiety.

Author, year	Disease stage	Treatment stage	Experimental design	Exercise prescription and sample	Adherence Attendance Compliance	Adverse events	Outcomes
Segal et al., 2003 [42]	I-IV	ADT	155 randomised RT vs. UC	**Resistance training** *n* = 82, 3 sessions per week for 12 weeks performing 2 sets of 8–12 reps at 60–70% of 1-RM	Adh: 91.2% Att: 79% Comp: NR	NR	FACT-F;FACT-P
Segal et al., 2009 [43]	I-IV;Gleason Score: 6.7 ± 0.9	Radiotherapy; Radiotherapy plus ADT	121 randomised RT vs. AT vs. UC	**Resistance training** *n* = 40, 3 sessions per week for 24 weeks performing 2 sets of 8–12 reps at 60–70% of 1-RM	Adh: NR Att: 88.0% Comp: NR	In the RT group, 1 patient experienced chest pain during exercise.	FACT-F; FACT-G; FACT-P
Galvão et al., 2010 [6]	Localised and nodal metastases; Gleason Score: 7.3	ADT	57 randomised Combined resistance and aerobic training vs. UC	**Combined resistance and aerobic training** *n* = 29, 2 sessions per week for 12 weeks; RT: 2–4 sets of 6–12RM AT: 15–20 min at 65–80% HR Sessions conducted in small groups of participants	Adh: NR Att: 94.0% Comp: NR	No adverse events.	EORTC QLQ-C30; SF-36
Bourke et al., 2011 [44]	Gleason Score: 7.0 ± 1.1	ADT	50 randomised Lifestyle intervention (combined resistance and aerobic training, nutrition advice, and home-based AT) vs. UC	**Combined resistance and aerobic training** *n* = 25, 2 sessions per week for 12 weeks; AT: 30 min at 55–85% HR; RT: 2–4 sets	Adh: NR Att: 95.2% Comp: 87.0%	No adverse events.	FACT-F; FACT-G; FACT-P
Cormie et al., 2013 [45]	Gleason Score: 8.2	Patients with established bone metastatic disease previously treated with ADT, 11 had previous radiotherapy and 4 had previous surgery	20 randomised RT plus home-based AT vs. UC	**Resistance training** *n* = 10, 2 sessions per week for 12 weeks performing 2–4 sets of 8–12RM; Sessions conducted in small groups of participants	Adh: 70.0% Att: 83.0% Comp: 93.2%	No adverse events.	MFSI-SF; SF-36; BSI-18
Galvão et al., 2014 [7]	II-IV	Patients previously treated with ADT and radiation therapy	100 randomised Combined resistance and aerobic training plus home-based AT vs. physical activity material	**Combined resistance and aerobic training** *n* = 50, 2 sessions per week for 24 weeks RT: 2–4 sets of 6–12RM AT: 20–30 min at 70–85% HR; Sessions conducted in small groups of participants	Adh: NR Att: 77.0% Comp: NR	One participant with preexisting back pain elected to cease the exercise program, as did one patient with a preexisting knee injury.	SF-36
Bourke et al., 2014 [46]	NR	ADT	100 randomised Lifestyle intervention (combined resistance and aerobic training, nutrition advice, and home-based AT) vs. UC	**Combined resistance and aerobic training** *n* = 50, 2 sessions per week for 12 weeks; AT: 30 min at 55–75% of HR; RT: 2–4 sets of 8–12 reps at 60% of 1-RM	Adh: 94.0% Att: NR Comp: NR	No adverse events.	FACT-F; FACT-P
Winters-Stone et al., 2015 [47]	NR	ADT; Chemotherapy; Radiotherapy; Bone metastasis	51 randomised Impact + RT plus home-based AT vs. home-based AT and FLX	**Multimodal exercise program** *n* = 29, 2 sessions per week for 48 weeks; Impact: 50 two-footed jumps from the ground with weighted vests RT: 1–3 sets of 8–12RM	Adh: 90.0% Att: 83.0% Comp: NR	No adverse events.	Schwartz Cancer Fatigue Scale; SF-36
Cormie et al., 2015 [10]	Gleason Score: 7.5	ADT; Chemotherapy; Radiotherapy	63 randomised Combined resistance and aerobic training plus home-based AT vs. UC	**Combined resistance and aerobic training** *n* = 32, 2 sessions per week for 12 weeks; AT: 20–30 min at 70–85% HR; RT: 1–4 sets of 6–12RM; Sessions conducted in small groups of participants	Adh: NR Att: 96.2% Comp: NR	One participant from the exercise group withdrew from the intervention due to feeling too nauseous, dizzy and fatigued to attend the exercise sessions.	FACIT; SF-36; BSI-18
Livingston et al., 2015 [11]	I-III	Surgery; Surgery plus radiotherapy; Surgery plus radiotherapy and ADT; Radiotherapy; Radiotherapy plus ADT; Surgery plus ADT	147 randomised Multimodal exercise program plus home-based AT vs. UC	**Multimodal exercise program** *n* = 142, 2 sessions per week for 12 weeks; AT: 20 min at 40–70% HR; RT: 1–2 sets of 8–12 reps BAL: NR FLX: NR	Adh: 87% Att: 85% Comp: NR	NR	CES-D; MAX-PC
Nilsen et al., 2015 [48]	Intermediate- and high-risk based on PSA and primary tumour	Radiotherapy plus ADT; following ADT	58 randomised RT vs. UC	**Resistance training** *n* = 28, 3 sessions per week for 16 weeks performing 1–3 sets of 10RM on Mondays, 2–3 sets of 10 reps at 80–90% of 10RM on Wednesdays, and 2–3 sets of 6RM on Fridays.	Adh: NR Att: NR Comp: 85.0%	Three patients in the RT group discontinued the intervention due to pain, 2 due to the pain in the knee and 1 patient due to back pain.	EORTC QLQ-C30
Winters-Stone et al., 2016 [49]	NR	Patients following primary treatment other than hormone therapy and not currently undergoing radiation or chemotherapy	64 randomised Impact + RT vs. UC Sessions with patients and spouses training together	**Multimodal exercise program** *n* = 32, 2 sessions per week for 24 weeks; Impact: 8–15 repetitions with weighted vests RT: 8–15RM	Adh: NR Att: 78.0% Comp: 94.0%	No adverse events.	SF-36
Hojan et al., 2017 [50]	Gleason Score: 8.8 ± 1.9	ADT	72 randomised Combined resistance and aerobic training vs. UC	**Combined resistance and aerobic training** *n* = 36, 3 sessions per week for 12 weeks; AT: 30 min; RT: 2 sets of 8 reps at 70–75% of 1-RM Sessions conducted either with one participant at a time or in small groups of participants	Adh: NR Att: 86.0% Comp: NR	Three overuse injuries to the lower extremities were reported in the exercise group.	FACT-F; EORTC QLQ-C30; FACT-G
Taaffe et al., 2017 [8]	Localised and nodal metastases; Gleason Score: 7.8	ADT; ADT plus radiotherapy; ADT; ADT plus surgery	159 randomised Impact + RT vs. Combined resistance and AT plus home-based AT vs. Delayed AT	**Multimodal exercise program** *n* = 57, 2 sessions per week for 24 weeks; Impact: bounding, skipping, drop jumping, hopping, and leaping activities RT: 2–4 sets of 6–12RM **Combined resistance and aerobic training** *n* = 54, 2 sessions per week for 24 weeks; AT: 20–30 min at 60–85% HR RT: 2–4 sets of 6–12RM Sessions conducted in small groups of participants	Adh: NR Att: 65.0 and 69.0% Comp: NR	Two men in Impact + RT withdrew due to compressed spinal discs and shoulder issues. Two men in Combined resistance and aerobic training had cardiovascular problems, with 1 requiring heart bypass surgery while another participant in ART developed back pain.	EORTC QLQ-C30; SF-36
Galvão et al., 2018 [51]	Patients with established bone metastatic disease	ADT; Prostatectomy; Radiotherapy; Brachytherapy; Chemotherapy	57 randomised Multimodal exercise program vs. UC	**Multimodal exercise program** *n* = 28, 3 sessions per week for 12 weeks; RT: 2 sets of 10–12RM AT: 20–30 min at 60–85% HR FLX: 2–4 reps for 30–60 s Sessions conducted in small groups of participants	Adh: NR Att: 89.0% Comp: NR	No adverse events.	FACIT; SF-36
Dawson et al., 2018 [52]	Including bone and nodal metastases Gleason Score: 7.5	ADT; Radiotherapy; Surgery; Chemotherapy	37 randomised RT vs. home-based FLX Part of the sample received whey protein isolate (~50%)	**Resistance training** *n* = 16, 3 sessions per week for 12 weeks performing 3 sets of 8–15 reps at 60–83% of 1-RM	Adh: 77.0% Att: 93.8% Comp: 88.3%	No adverse events.	BFI; FACT-G; FACT-P; CES-D
Alibhai et al., 2019 [53]	Gleason score range from 6 to 10	ADT	53 randomised Personally supervised vs. group supervised vs. home-based exercise program	**Multimodal exercise program** *n* = 19, 3 sessions per week for 24 weeks Sessions conducted with one participant at a time	Adh: NR Att: 75.0% Comp: NR	One adverse event in the multimodal exercise program	FACT-F; FACT-G; FACT-P
Ndjavera et al., 2019 [54]	Locally advanced and metastatic patients; Gleason score range from 6 to 10	ADT; ADT plus radiotherapy	50 randomised Combined resistance and aerobic training plus home-based AT and RT vs. UC	**Combined resistance and aerobic training** *n* = 24, 2 sessions per week for 12 weeks; AT: 6 bouts of 5 min at 55–85% HR RT: 2–4 sets of 10 reps	Adh: NR Att: 70.0% Comp: NR	No adverse events.	FACIT; FACT-P

*1-RM* 1-repetition maximum, *Adh* adherence, *ADT* androgen deprivation therapy, *AT* aerobic training, *Att* attendance, *BAL* balance exercises, *BFI* Brief Fatigue Inventory, *BSI-18* Brief Symptom Inventory-18, *CES-D* Center for Epidemiologic Studies—Depression Scale, *Comp* Compliance, *EORTC QLQ-C30* European Organisation for Research and Treatment of Cancer Quality of Life Questionnaire C30, *FACIT* Functional Assessment of Chronic Illness Therapy, *FACT-F* Functional Assessment of Cancer Therapy—Fatigue, *FACT-G* Functional Assessment of Cancer Therapy—General, *FACT-P* Functional Assessment of Cancer Therapy—Prostate, *FLX* flexibility training, *GnRH* Gonadotrophin-releasing hormone, *MAX-PC* Memorial Anxiety Scale for Prostate Cancer, *MFSI-SF* Multidimensional Fatigue Symptom Inventory-Short Form, *NR* not reported, *RT* resistance training, *SF-36* 36-Item Short-Form Health Survey, *UC* usual care control group.

The mean exercise intervention duration was 17.3 ± 9.5 weeks with either 2 [refs. 6–8, 10, 11, 44–47, 49, 54] or 3. sessions per week [42, 43, 48, 50–53] (average: 2.5 ± 0.8 sessions per week). The average total prescribed resistance training volume was 8334 ± 4568 repetitions with a weekly training volume of 489 ± 195 repetitions which is equivalent to a training volume of 197 ± 58 repetitions per session (~2 sets of 10 repetitions for 10 exercises). In addition, the highest peak intensity reached throughout the resistance training programs was 85% [6–8, 11, 48], followed by 83% [52], 80% [45, 47, 49], 75% [50, 51], 70% [42, 43] and 60% [46] of 1-RM. Information about resistance training volume was not reported by two studies [44, 53], while four studies [11, 44, 53, 54] did not report intensity. Exercise program adherence ranged from 70 to 94% (reported in 6 of 18 studies) [11, 42, 45–47, 52], while the attendance and compliance ranged from 65 to 94% (reported in 16 of 18 studies) [6–8, 10, 11, 42–45, 47, 49–54] and from 70 to 94% (reported in 5 of 18 studies) [44, 45, 49, 50, 53], respectively. Supervised exercise sessions were conducted in small groups of participants in seven studies [6–8, 10, 45, 50, 51], while one study reported that exercise sessions were conducted with one participant at a time [53]. Adverse events related to the exercise interventions were identified in seven studies [7, 8, 11, 43, 49, 51, 54], while nine studies [6, 44–47, 49, 51, 52, 54] reported no adverse events during the intervention period, and 2 [refs. 11, 42] did not report this information.

### Risk of bias assessment

For the primary outcome of fatigue, 7.1% of the studies presented some concern in risk of bias assessment (1 of 14 studies) [53]. The concern with the fatigue assessment was due to the selection of the reported result as one study did not report sufficient information for the outcome at baseline (1 of 14, 7.1%; *some concern*) [53]. For the secondary outcomes, there was some concern of bias observed in quality of life (13.3%, 2 of 15 studies) [11, 53], depression (25.0%, 1 of 4 studies) [11] and for the anxiety assessment (33.3%, 1 of 3 studies) [11]. The risk of bias assessment for each outcome is shown in Table 3, while the individual assessment is shown in Supplementary Material Fig. S1.

**Table 3:** Risk of bias of included studies.

Outcome	Randomisation process	Deviation from intended interventions	Missing outcome data	Measurement of the outcome	Selection of the reported result	Overall bias
Fatigue, *n* = 14						
Low risk	14 (100%)	14 (100%)	14 (100%)	14 (100%)	13 (92.9%)	13 (92.9%)
Some concerns	0	0	0	0	1 (7.1%)	1 (7.1%)
High risk	0	0	0	0	0	0
Quality of life, *n* = 15						
Low risk	14 (93.3%)	15 (100%)	15 (100%)	15 (100%)	14 (93.3%)	13 (86.7%)
Some concerns	1 (6.7%)	0	0	0	1 (6.7%)	2 (13.3%)
High risk	0	0	0	0	0	0
Depression, *n* = 4						
Low risk	3 (75.0%)	4 (100%)	4 (100%)	4 (100%)	4 (100%)	3 (75.0%)
Some concerns	1 (25.0%)	0	0	0	0	1 (25.0%)
High risk	0	0	0	0	0	0
Anxiety, *n* = 3						
Low risk	2 (66.7%)	3 (100%)	3 (100%)	3 (100%)	3 (100%)	2 (66.7%)
Some concerns	1 (33.3%)	0	0	0	0	1 (33.3%)
High risk	0	0	0	0	0	0

*n* number of studies.

### Exercise effects on fatigue

**Fig. 2:**
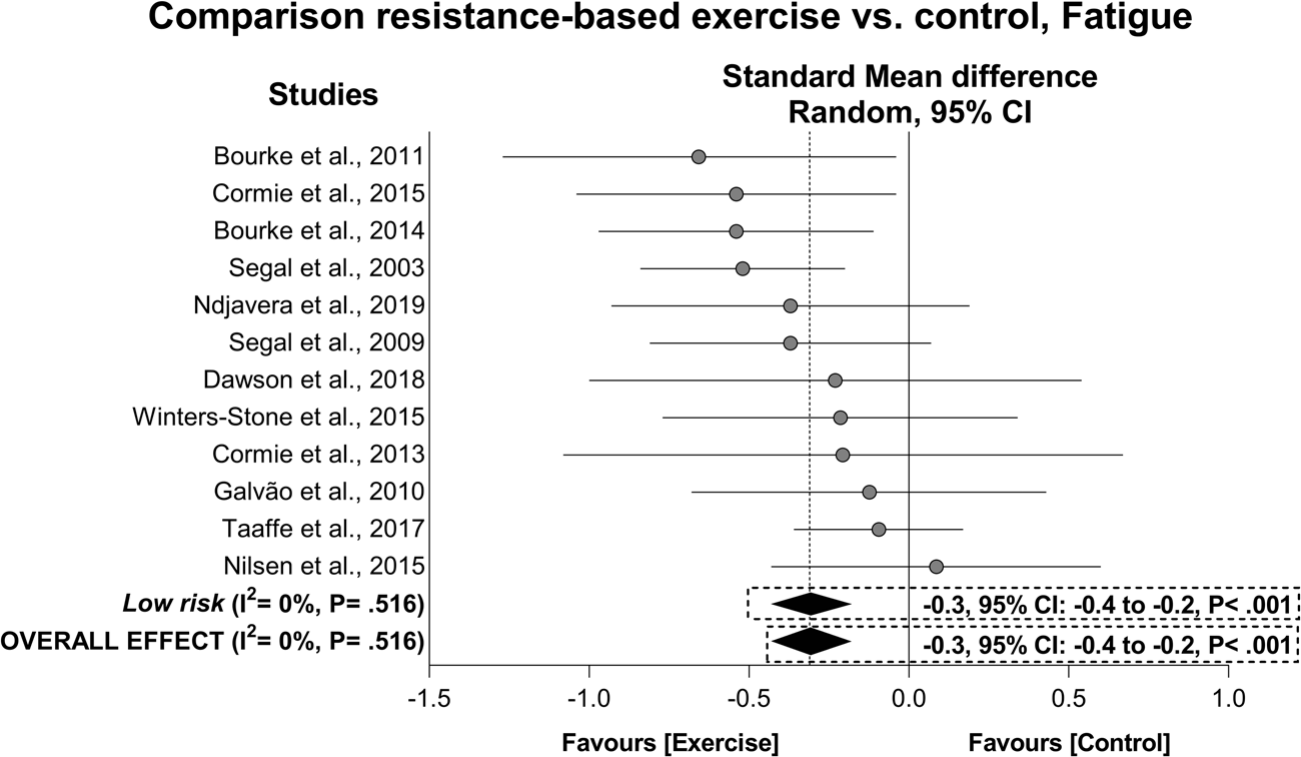
Mean difference effects of resistance-based exercise compared with control on fatigue. Overall and subgroup analyses conducted with a random-effects model. Grey and white circles represent study specific estimates based on risk of bias assessment (Low risk, and some concern or high risk of bias, respectively); diamonds represent pooled estimates of random-effects meta-analysis.

For fatigue, exercise resulted in a significant overall mean ES of −0.3 (95% CI: −0.4 to −0.2, *P* < 0.001) with I^2^ = 0% in 507 prostate cancer patients who undertook exercise interventions compared to 459 patients in control groups (Fig. 2). The result was maintained in the subgroup analysis involving the low risk studies. For questionnaires used to assess patient-reported fatigue, positive effects of −5.2 pts (95% CI: −10.1 to −0.2 pts, *P* = 0.040) were found in the EORTC QLQ-C30_Fatigue_ with I^2^ = 31%, and 3.9 pts (95% CI: 2.6 to 5.3 pts, *P* < 0.001) with I^2^ = 0% in the FACT-F. When low risk subgroup analyses were undertaken, all effects were maintained. There were insufficient data for the MFSI-SF, FACIT, BFI and Schwartz Cancer Fatigue Scale for further analysis. Hojan et al. [50] was considered an outlier in the overall and subgroup analysis for fatigue and for FACT-F being omitted from the abovementioned results. No publication bias was found (*P* = 0.327–0.455). Main effects along with sensitivity and publication bias adjusted results are presented in Table 4. The exercise effect in patients presenting with high mean fatigue levels [54] was slightly greater than that overall observed in overall fatigue (ES = −0.4, 95% CI: −0.9 to 0.2).

**Table 4:** Overall intervention effects on the patient-reported outcomes in prostate cancer patients.

Outcomes	Analysis	*n*	Sample	Mean difference	95% CI	I^2^	Mean difference *P* value
Fatigue							
Overall^a^	All^b^	12	896	−0.3	−0.4 to −0.2	0%	<0.001
	Low risk^b^	12	896	−0.3	−0.4 to −0.2	0%	<0.001
FACT-F, pts^b^	All^b^	6	474	3.9	2.6 to 5.3	0%	<0.001
	Low risk^b^	4	365	4.1	2.8 to 5.4	0%	<0.001
EORTC QLQ-C30_Fatigue_	All	5	396	−5.2	−10.1 to −0.2	31%	0.040
	Low risk	5	396	−5.2	−10.1 to −0.2	31%	0.040
MFSI-SF	All^d^	1	20	−4.2	−17.6 to 9.2	–	–
	Low risk^d^	1	20	−4.2	−17.6 to 9.2	–	–
FACIT-Fatigue	All^d^	2	113	3.6	1.2 to 6.0	–	–
	Low risk^d^	2	113	3.6	1.2 to 6.0	–	–
BFI	All^d^	1	26	−0.8	−2.6 to 1.0	–	–
	Low risk^d^	–	–	–	–	–	–
Schwartz Cancer Fatigue Scale	All^d^	1	51	5.5	−3.1 to 14.0	–	–
	Low risk^d^	1	51	5.5	−3.1 to 14.0	–	–
Quality of life							
Overall^a^	All^b^	12	846	0.2	0.0 to 0.4	28%	0.014
	Low risk^b^	11	716	0.2	0.0 to 0.4	32%	0.018
FACT-G	All^b^	4	187	4.4	1.7 to 7.2	0%	0.002
	Low risk^b^	3	150	4.7	1.7 to 7.8	0%	0.002
FACT-P	All^b^	6	396	4.8	3.1 to 6.5	12%	<0.001
	Low risk^b^	5	359	6.3	3.8 to 8.7	0%	<0.001
SF-36_Physical health composite_	All	5	293	0.8	−0.7 to 2.3	45%	0.291
	Low risk	5	293	0.8	−0.7 to 2.3	45%	0.291
SF-36_Physical functioning_	All^b^	5	287	1.9	0.7 to 3.2	0%	0.003
	Low risk	5	287	1.9	0.7 to 3.2	0%	0.003
SF-36_Role physical_	All	4	239	2.2	0.3 to 4.2	0%	0.025
	Low risk	4	239	2.2	0.3 to 4.2	0%	0.025
SF-36_Bodily pain_	All	4	239	−0.2	−2.5 to 2.1	16%	0.843
	Low risk	4	239	−0.2	−2.5 to 2.1	16%	0.843
SF-36_General health_	All	4	239	1.8	−0.5 to 4.0	30%	0.131
	Low risk	4	239	1.8	−0.5 to 4.0	30%	0.131
SF-36_Mental health composite_	All^b^	4	239	2.9	1.2 to 4.7	0%	0.001
	Low risk^b^	4	239	2.9	1.2 to 4.7	0%	0.001
SF-36_Vitality_	All^b,c^	6	450	0.2	−0.7 to 1.1	21%	0.655
	Low risk^b^	6	450	0.2	−0.7 to 1.1	21%	0.655
SF-36_Social functioning_	All	4	239	4.7	2.7 to 6.6	0%	<0.001
	Low risk	4	239	4.7	2.7 to 6.6	0%	<0.001
SF-36_Role emotional_	All	4	239	0.9	−1.0 to 2.8	0%	0.371
	Low risk	4	239	0.9	−1.0 to 2.8	0%	0.371
SF-36_Mental Health_	All	4	239	2.4	0.8 to 4.0	0%	0.004
	Low risk	4	239	2.4	0.8 to 4.0	0%	0.004
EORTC QLQ-C30_Global_	All	4	310	−0.1	−5.1 to 4.9	44%	0.980
	Low risk	3	180	−1.3	−8.5 to 6.0	50%	0.731
EORTC QLQ-C30_Physical functioning_	All^c^	4	310	1.9	−0.6 to 4.5	35%	0.138
	Low risk	3	180	3.1	−0.3 to 6.4	27%	0.073
EORTC QLQ-C30_Role functioning_	All	4	310	5.0	−0.6 to 10.6	44%	0.080
	Low risk	3	106	8.5	−0.2 to 17.3	45%	0.057
EORTC QLQ-C30_Emotional functioning_	All^b^	3	180	6.1	1.0 to 11.2	5%	0.020
	Low risk	3	180	6.1	1.0 to 11.2	5%	0.020
EORTC QLQ-C30_Cognitive functioning_	All	4	310	4.9	1.7 to 8.1	39%	0.003
	Low risk	3	180	6.2	0.5 to 11.9	29%	0.034
EORTC QLQ-C30_Social functioning_	All	4	310	3.4	−1.7 to 8.4	0%	0.190
	Low risk	3	180	3.5	−2.9 to 10.0	0%	0.282
EORTC QLQ-C30_Nausea and vomiting_	All	3	180	−1.8	−4.1 to 0.5	14%	0.128
	Low risk	3	180	−1.8	−4.1 to 0.5	14%	0.128
EORTC QLQ-C30_Pain_	All	3	180	−4.3	−11.6 to 3.1	21%	0.258
	Low risk	3	180	−4.3	−11.6 to 3.1	21%	0.258
EORTC QLQ-C30_Dyspnoea_	All	3	180	−8.8	−16.0 to −1.6	0%	0.016
	Low risk	3	180	−8.8	−16.0 to −1.6	0%	0.016
EORTC QLQ-C30_Insomnia_	All	3	180	−5.0	−15.6 to 5.6	32%	0.358
	Low risk	3	180	−5.0	−15.6 to 5.6	32%	0.358
EORTC QLQ-C30_Appetite loss_	All	3	180	−0.5	−4.5 to 3.4	6%	0.789
	Low risk	3	180	−0.5	−4.5 to 3.4	6%	0.789
EORTC QLQ-C30_Constipation_	All	3	180	1.5	−3.7 to 6.8	0%	0.567
	Low risk	3	180	1.5	−3.7 to 6.8	0%	0.567
EORTC QLQ-C30_Diarrhoea_	All	3	180	0.8	−9.3 to 10.8	56%	0.878
	Low risk	3	180	0.8	−9.3 to 10.8	56%	0.878
EORTC QLQ-C30_Finance_	All^d^	2	107	−0.9	−7.7 to 5.9	–	–
	Low risk^d^	2	107	−0.9	−7.7 to 5.9	–	–
Depression							
Overall^a^	All	4	239	−0.2	−0.5 to 0.0	0%	0.091
	Low risk	3	109	−0.3	−0.7 to 0.0	0%	0.077
CES-D	All^d^	2	156	−1.9	−3.8 to −0.1	–	–
	Low risk^d^	1	26	−2.8	−8.9 to 3.3	–	–
BSI-18_Depression_	All^d^	2	83	−1.2	−2.1 to −0.2	–	–
	Low risk^d^	2	83	−1.2	−2.1 to −0.2	–	–
Anxiety							
Overall^a^	All	3	212	−0.3	−0.5 to 0.0	0%	0.071
	Low risk^d^	2	83	−0.1	−0.5 to 0.3	–	–
BSI-18_Anxiety_	All^d^	2	83	−0.5	−1.2 to 0.2	–	–
	Low risk^d^	2	83	−0.5	−1.2 to 0.2	–	–
MAX-PC	All^d^	1	129	2.5	0.4 to 4.6	–	–
	Low risk^d^	–	–	–	–	–	–

Questionnaires reverse scaled (High scores—Less fatigue).

*BFI* Brief Fatigue Inventory, *BSI-18* Brief Symptom Inventory-18, *CES-D* Center for Epidemiologic Studies—Depression Scale, *EORTC QLQ-C30* European Organisation for Research and Treatment of Cancer Quality of Life Questionnaire C30, *FACIT* Functional Assessment of Chronic Illness Therapy, *FACT-F* Functional Assessment of Cancer Therapy—Fatigue, *FACT-G* Functional Assessment of Cancer Therapy—General, *FACT-P* Functional Assessment of Cancer Therapy—Prostate, *MAX-PC* Memorial Anxiety Scale for Prostate Cancer, *MFSI-SF* Multidimensional Fatigue Symptom Inventory-Short Form, *n* number of comparisons, *SF-36* 36-Item Short-Form Health Survey.

^a^Analysis performed using standardized mean difference effect.

^b^Adjustment after sensitivity analysis omitting one study at a time.

^c^Trim-and-fill adjustment after significant effect of publication bias in Egger’s test (*P* < 0.1).

^d^Insufficient data for analysis.

In the dose-response analysis, the meta-regression models did not present significant associations between mode (i.e. resistance training alone vs. resistance-based exercise programs), duration (ranging from 8 to 60 weeks), resistance training weekly volume (ranging from 320 to 975 repetitions) and peak intensity (ranging from 60 to 85% of 1-RM) with patient-reported fatigue (univariate: *P* = 0.055–0.988, multivariate: r^2^ = 100%, *P* = 0.375, Table 5).

**Table 5:** Univariable and multivariable meta-regression on fatigue and quality of life mean differences and resistance training mode, duration, weekly volume and peak intensity.

Outcomes	RT components	Range	Univariate		Multivariate		
			Coef ± SE (95% CI)	*P* value	Coef ± SE (95% CI)	*P* value	Model
Fatigue^a^	Mode, RT alone or combined	RT alone/RT combined	−0.0 ± 0.2 (−0.4 to 0.4)	0.988	−0.2 ± 0.4 (−1.2 to 0.9)	0.672	r^2^ = 100% I^2^ = 0% *P* = 0.375
	Training duration, wk	8 to 60	0.01 ± 0.01 (−0.01 to 0.03)	0.215	−0.01 ± 0.01 (−0.02 to 0.03)	0.404	
	RT weekly volume, reps	320 to 975	0.0 ± 0.0 (−0.001 to 0.001)	0.659	0.0 ± 0.0 (−0.002 to 0.003)	0.689	
	RT intensity, 1-RM	60 to 85%	0.02 ± 0.01 (−0.001 to 0.04)	0.055	−0.03 ± 0.02 (−0.02 to 0.08)	0.186	
Quality of life^b^	Mode, RT alone or combined	RT alone/RT combined	−0.2 ± 0.2 (−0.6 to 0.2)	0.262	0.2 ± 0.4 (−0.9 to 1.3)	0.630	r^2^ = −6.8%^c^ I^2^ = 40% *P* = 0.509
	Training duration, wk	8 to 60	−0.02 ± 0.02 (−0.1 to 0.0)	0.322	−0.02 ± 0.02 (−0.1 to 0.03)	0.374	
	RT weekly volume, reps	240 to 975	0.0 ± 0.0 (−0.001 to 0.001)	0.306	0.001 ± 0.001 (−0.0 to 0.0)	0.393	
	RT intensity, 1-RM	70 to 85%	−0.03 ± 0.01 (−0.06 to 0.01)	0.096	−0.02 ± 0.02 (−0.1 to 0.02)	0.286	

*1-RM* 1-repetition maximum, *95% CI* 95% confidence intervals, *Coef* Meta-regression coefficient, *RT* resistance training, *SE* standard error, *wk* weeks.

^a^Assessed combining the following instruments: the Functional Assessment of Cancer Therapy—Fatigue (FACT-F), European Organisation for Research and Treatment of Cancer Quality of Life Questionnaire C30—Fatigue (EORTC QLQ-C30_Fatigue_), Multidimensional Fatigue Symptom Inventory-Short Form (MFSI-SF), Functional Assessment of Chronic Illness Therapy (FACIT), Brief Fatigue Inventory (BFI), and the Schwartz Cancer Fatigue Scale questionnaires.

^b^Assessed combining the following instruments: The Functional Assessment of Cancer Therapy—Prostate (FACT-P) and—General (FACT-G), the 36-Item Short-Form Health Survey (SF-36) physical and mental health composite and EORTC QLQ-C30—Global.

^c^Analogous to r^2^ = 0%.

### Exercise effects on quality of life

**Fig. 3:**
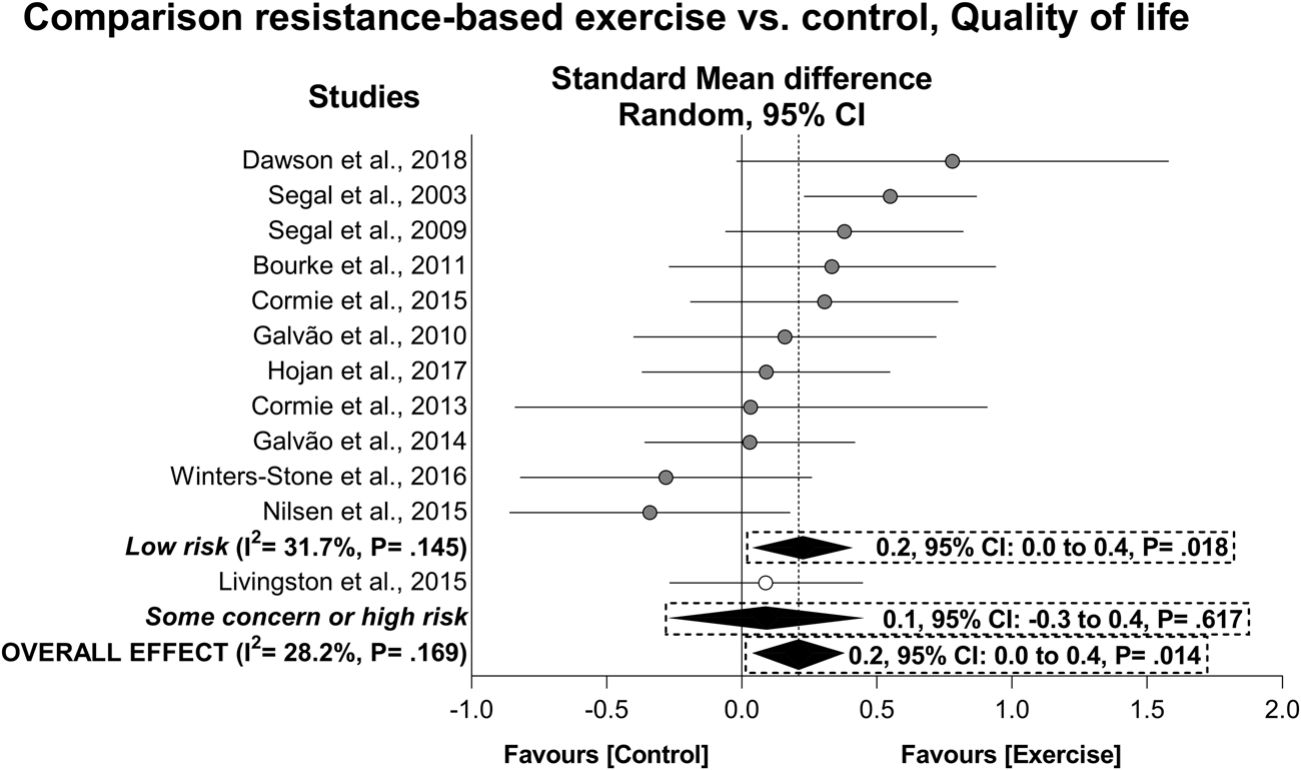
Mean difference effects of resistance-based exercise compared with control on quality of life. Overall and subgroup analyses conducted with a random-effects model. Grey and white circles represent study specific estimates based on risk of bias assessment (Low risk, and some concern or high risk of bias, respectively); diamonds represent pooled estimates of random-effects meta-analysis.

A significant improvement was observed in the quality of life (ES = 0.2, 95% CI: 0.0 to 0.4, *P* = 0.014) in a sample of 447 prostate cancer patients who undertook resistance-based exercise compared to 399 patients in control groups (Fig. 3). This result was maintained after low risk subgroup analysis (Table 4). Exercise also resulted in a positive effect of 4.4 pts (95% CI: 1.7 to 7.2 pts, *P* = 0.002) in FACT-G and 4.8 pts (95% CI: 3.1 to 6.5 pts, *P* < 0.001) in the FACT-P with I^2^ = 0 and 12%, respectively. Low risk subgroup analysis indicated a positive effect of exercise on FACT-P and FACT-G. In addition, exercise resulted in significant effects in specific components of the SF-36 (i.e. physical functioning, role physical, mental health composite, social functioning and mental health, *P* = 0.003–0.020) as indicated by the low risk subgroup analyses, while effects on the EORTC QLQ-C30 components (i.e. emotional functioning, cognitive functioning, and dyspnoea; *P* = 0.003–0.020) were observed in the overall and maintained in the subgroup analysis. Outliers were identified in FACT-G [50], FACT-P [43], SF-36_Physical functioning_ and SF-36_Mental health composite_ [49], SF-36_Vitality_ [6] and EORTC QLQ-C30_Emotional functioning_ [11] analyses and omitted from the abovementioned results. No publication bias was observed (*P* = 0.272–0.539). The main effects along with sensitivity and publication bias adjusted results are presented in Table 4. The exercise effects in patients presenting with low mean quality of life values [50] were somewhat smaller than that overall observed in overall quality of life (ES = 0.1, 95% CI: −0.4 to 0.6).

In the dose-response analysis, the meta-regression models did not present significant associations between mode (i.e. resistance training alone vs. resistance-based exercise programs), duration (ranging from 8 to 60 weeks), resistance training weekly volume (ranging from 240 to 975 repetitions) and peak intensity (ranging from 70 to 85% of 1-RM) with effects on patient-reported quality of life (univariate: *P* = 0.096–0.322, multivariate: r^2^ = −6.8%, *P* = 0.509, Table 5).

### Exercise effects on depression and anxiety symptoms

**Fig. 4:**
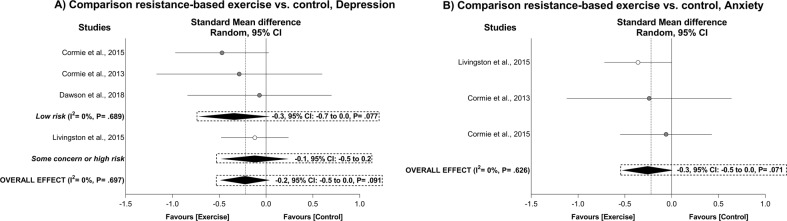
Mean difference effects of resistance-based exercise compared with control on depression (A) and anxiety (B). Overall and subgroup analyses conducted with a random-effects model. Grey and white circles represent study specific estimates based on risk of bias assessment (Low risk, and some concern or high risk of bias, respectively); diamonds represent pooled estimates of random-effects meta-analysis.

There was no significant exercise effect for overall depression and overall anxiety (ES = −0.2, 95% CI: −0.5 to 0.0. *P* = 0.091 and ES = −0.3, 95% CI: −0.5 to 0.0, *P* = 0.071, respectively; Table 4 and Fig. 4). The heterogeneity was I^2^ = 0% with no effect of publication bias (*P* = 0.717–0.815). Effects on overall depression were maintained in the subgroup analysis, while not conducted on anxiety given the small number of studies included. There were insufficient data for the dose-response analysis in depression and anxiety outcomes.

## Discussion

The present review examined the resistance training effect and dose-response on common patient-reported outcomes in prostate cancer patients. The main findings were: (1) supervised resistance exercise produced significant positive benefits on overall fatigue and quality of life whether undertaken as a sole exercise mode or combined with other exercise modes; and (2) the beneficial effects on fatigue and quality of life were independent of the prescribed exercise volume and intensity undertaken. In addition, exercise effects on overall depression and anxiety outcomes were positive, although not significant. These results are clinically relevant and demonstrate the potential to use a lower weekly volume and moderate intensity exercise as a strategy to improve quality of life and reduce cancer-related fatigue during and following active treatment.

Fatigue is one of the most reported symptoms in prostate cancer patients [3]. Considering its multifactorial nature, fatigue is examined via physical, social, emotional and functional wellbeing aspects in several questionnaires. In the present review, a positive exercise effect was indicated for overall fatigue and there were increases beyond the minimally important clinically difference in the FACT-F and EORTC QLQ-C30_Fatigue_, a result sustained in the low risk randomised controlled trials. However, it is important to note that patients included in the present analysis generally presented low baseline levels of fatigue which may indicate that patients with greater fatigue may derive a greater benefit from exercise [8]. Previous studies [8, 15, 55] are in accordance with the present analysis, demonstrating changes of ~3 pts in the EORTC QLQ-C30_Fatigue_ [8] moderated by higher baseline fatigue levels [8, 55]. Thus, the present findings suggest that supervised resistance-based programs can reduce levels of fatigue in prostate cancer patients at various disease stages and it may well be that patients with high baseline levels of fatigue may experience greater improvements as previously reported [8, 55]. Furthermore, the lack of association between resistance training dosage and its effects on overall fatigue also indicates the potential of exercise medicine using a lower exercise dosage to manage cancer-related fatigue. The benefits to using a low dosage of resistance training (e.g. less repetitions per exercise at a moderate to high intensity) appears to be regardless of the intervention duration as short-term programs (8 weeks) produced similar reductions in overall fatigue to that achieved by longer duration interventions (e.g. 60 weeks) in prostate cancer patients at different treatment stages. Finally, our results also demonstrate similar effects between different exercise modes as denoted by the nonsignificant univariate meta-regression model. This result agrees with a previous study [56] showing that resistance, aerobic or combined resistance and aerobic exercise promotes similar effects on cancer-related fatigue in cancer patients (−0.21, −0.23 and −0.26 SMD, respectively) and demonstrates the potential use of a low dosage even when resistance exercise is prescribed as a single mode of exercise in men with prostate cancer.

Overall quality of life was significantly improved in prostate cancer patients who undertook supervised resistance-based exercise programs. Our results are in accordance with meta-analyses undertaken in healthy older adults [57] and those with cancer [58, 59] where quality of life was assessed using the SF-36 and FACT questionnaires. Furthermore, a low resistance training dosage may culminate in comparable effects to that achieved by higher dosages in quality of life as noted by the nonsignificant meta-regression models. These results concur with those of Sweegers et al. [59] who found no difference in quality of life in cancer patients with varying weekly exercise volumes and energy expenditures during or following treatment. Importantly, the present findings suggest that less repetitions per exercise at a moderate to high intensity is sufficient to improve quality of life in prostate cancer patients, which is smaller than the dosage currently proposed for this outcome [12]. Therefore, these results are of importance for prostate cancer patients in different treatment phases as it reduces the time required (and effort/energy expenditure required) for exercise, which may permit higher attendance and compliance along with sustained benefits in longer term exercise programs.

The present analysis essentially included a small number of studies involving non-depressed men with prostate cancer as observed by the baseline values reported [10, 11, 45, 52]. Despite comparable effects to those observed in fatigue and quality of life, the exercise effects on depression symptoms only approached statistical significance, presenting similar effect sizes to studies in other cancer populations [60] but smaller than that observed when different clinical populations are pooled [61]. As a result, despite the overall significant association between resistance exercise and reduced depressive symptoms observed previously [60, 61], larger and more rigorous studies are necessary to clarify the effect of resistance-based exercise on depression in men with prostate cancer and include patients with existing depression. In addition, whether combining resistance and aerobic exercise accrues superior effects than resistance training alone is yet to be determined.

The use of exercise medicine is important for prostate cancer patients given the anxieties associated living with a cancer diagnosis and fears regarding its progression [4, 5]. We did not observe significant anxiolytic effects of resistance-based exercise programs in prostate cancer patients, which contrasts with studies in older adults [62], breast cancer patients [63] and other groups of cancer patients [64]. The reasons for such differences could be related to the few studies included in the analysis and apparently low baseline anxiety values of men in these studies. However, given this outcome approached statistical significance (*P* = 0.071), it is possible to suggest that exercise may in some cases counter anxiety, especially in those patients with greater anxiety levels. This result is important given the relatively high prevalence of this symptom across the treatment spectrum [5], and the association of anxiety with poorer surgical outcomes [65]. Moreover, the number of studies included also precluded further analysis regarding the exercise minimal dosage or mode. Thus, it is not possible to examine if low dosages or the prescription of resistance training alone may provide meaningful effects in this outcome as observed in fatigue and quality of life, or even in other types of cancer [63, 64].

The strengths of the present study include the large number of trials and participants assessing different patient-report outcomes, and the assessment of low risk studies. However, there are also some limitations which are worthy of comment. First, there were insufficient data to perform dose-response analyses on the depression and anxiety outcomes. These were both secondary outcomes in our analysis and in all exercise trials where the recruitment was not based on depression or anxiety levels. Studies designed to directly investigate these outcomes in those with depression and anxiety are required to determine the efficacy of exercise and its dosage in this patient/survivorship group. Second, we used prescribed instead of the complied exercise dosage given that this is predominantly reported in the studies analysed. However, reporting of complied dosage [12, 66] in future studies will assist with better defining the upper and lower exercise prescription thresholds. Third, the nature of supervised group sessions may be considered a potential factor in some of the included studies. Participants in supervised exercise group sessions are likely to share experiences related to treatment as well as develop camaraderie during the exercise program [8] and, consequently, this may contribute to additional benefits in these individual studies. Lastly, although most patients included were on ADT, the lower heterogeneity within the analyses may indicate that the response is quite similar following treatment.

Establishing the minimal-dose approach to enhance patient-reported outcomes is challenging in the field of exercise oncology. As far as are aware, this is the first study to examine the resistance training dose-response on patient-report outcomes in prostate cancer patients. Our findings suggest that a low dosage (e.g. less repetitions per exercise undertaken at a moderate to high intensity), less than that proposed in the latest exercise guideline for cancer patients [12], is sufficient to induce meaningful benefits for fatigue and quality of life in patients during or following active treatment. Furthermore, the present results regarding depression and anxiety outcomes are also promising, indicating the potential use of resistance-based training to avoid further psychological distress during and following active treatment.

## Supplementary information

Supplementary material
