# Supplementary material for: What is the minimal dose for resistance exercise effectiveness in prostate cancer patients? Systematic review and meta-analysis on patient-reported outcomes
Source: Prostate Cancer Prostatic Dis. 2020 Nov 20;24(2):465–81. doi: 10.1038/s41391-020-00301-4 (PMC8134054; doi:10.1038/s41391-020-00301-4)
Supplement: Supplementary file 1 — Supplementary material [file 41391_2020_301_MOESM1_ESM.docx]

**What is the minimal dose for resistance exercise effectiveness in prostate cancer patients? Systematic review and meta-analysis on patient-reported outcomes.**

**Electronic Supplementary Material table headings.**

* Electronic Supplementary Material Table S1. Literature search strategy used for the PubMed database

* Electronic Supplementary Material Figure S1. Individual risk of bias assessment at outcome level for A) fatigue, B) quality of life, C) depression and D) anxiety. Green circles, low risk; yellow circles, some concerns; red circles, high risk of bias.

**Table S1**. Literature search strategy used for the PubMed database

| #1” Search “prostate cancer”[Mesh] OR Prostate Neoplasms [title/abstract] OR Neoplasms, Prostate [title/abstract] OR Neoplasm, Prostate [title/abstract] OR Prostate Neoplasm [title/abstract] OR Neoplasms, Prostatic [title/abstract] OR Neoplasm, Prostatic [title/abstract] OR Prostatic Neoplasm [title/abstract] OR Prostate Cancer [title/abstract] OR Cancer, Prostate [title/abstract] OR Cancers, Prostate [title/abstract] OR Prostate Cancers [title/abstract] OR Cancer of the Prostate [title/abstract] OR Prostatic Cancer [title/abstract] OR Cancer, Prostatic [title/abstract] OR Cancers, Prostatic [title/abstract] OR Prostatic Cancers [title/abstract] OR Cancer of Prostate [title/abstract]  #2” Search “resistance training”[Mesh] OR Training, Resistance [title/abstract] OR Strength Training [title/abstract] OR Training, Strength [title/abstract] OR Weight-Lifting Strengthening Program [title/abstract] OR Strengthening Program, Weight-Lifting [title/abstract] OR Strengthening Programs, Weight-Lifting [title/abstract] OR Weight Lifting Strengthening Program [title/abstract] OR Weight-Lifting Strengthening Programs [title/abstract] OR Weight-Lifting Exercise Program [title/abstract] OR Exercise Program, Weight-Lifting [title/abstract] OR Exercise Programs, Weight-Lifting [title/abstract] OR Weight Lifting Exercise Program [title/abstract] OR Weight-Lifting Exercise Programs [title/abstract] OR Weight-Bearing Strengthening Program [title/abstract] OR Strengthening Program, Weight-Bearing [title/abstract] OR Strengthening Programs, Weight-Bearing [title/abstract] OR Weight Bearing Strengthening Program [title/abstract] OR Weight-Bearing Strengthening Programs [title/abstract] OR Weight-Bearing Exercise Program [title/abstract] OR Exercise Program, Weight-Bearing [title/abstract] OR Exercise Programs, Weight-Bearing [title/abstract] OR Weight Bearing Exercise Program [title/abstract] OR Weight-Bearing Exercise Programs [title/abstract].  #1 AND #2 |
| --- |


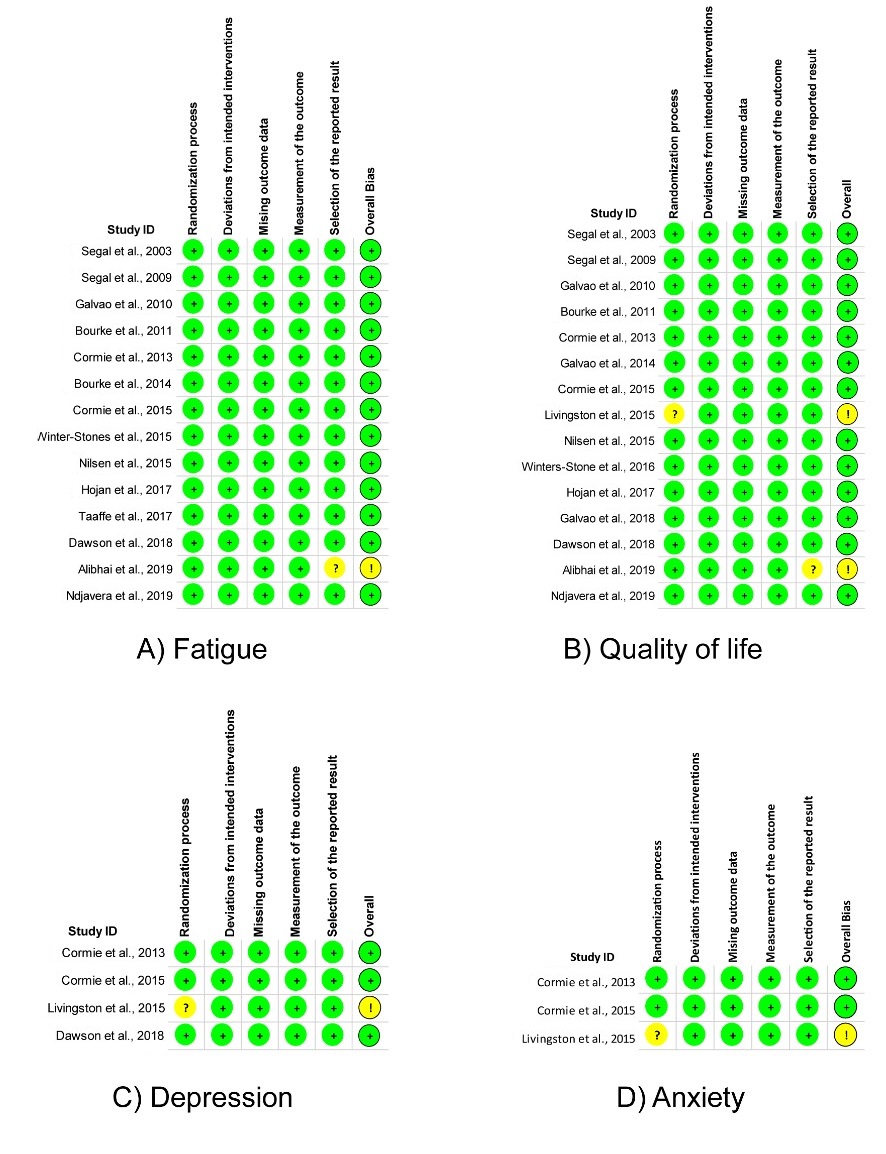


**Figure S1.** Individual risk of bias assessment at outcome level for A) fatigue, B) quality of life, C) depression and D) anxiety. Green circles, low risk; yellow circles, some concerns; red circles, high risk of bias.
